# Investigation of hybrid plasmons in a highly crystalline Bi_2_Se_3_/C_60_ heterostructure using low-loss electron energy loss spectroscopy

**DOI:** 10.1038/s43246-025-00886-0

**Published:** 2025-07-29

**Authors:** Mairi McCauley, Lida Ansari, Farzan Gity, Matthew Rogers, Joel Burton, Satoshi Sasaki, Quentin Ramasse, Craig Knox, Paul K. Hurley, Donald MacLaren, Timothy Moorsom

**Affiliations:** 1 SUPA, School of Physics and Astronomy, University of Glasgow, Glasgow, UK; 2 Micronano Electronics Group, Tyndall National Institute, University College Cork, Cork, Republic of Ireland; 3 School of Physics and Astronomy, University of Leeds, Leeds, UK; 4 SuperSTEM, SciTech Daresbury Science and Innovation Campus, Daresbury, UK; 5 School of Chemical and Process Engineering, University of Leeds, Leeds, UK; 6 School of Chemistry, University College Cork, Cork, Ireland

**Keywords:** Topological insulators, Carbon nanotubes and fullerenes

## Abstract

Topological Insulators (TIs) present an interesting materials platform for nanoscale, high frequency devices because they support high mobility, low scattering electronic transport within confined surface states. However, a robust methodology to control the properties of surface plasmons in TIs has yet to be developed. Surface doping of TIs with molecules may provide tunable control of the two-dimensional plasmons in Bi_2_Se_3_, but exploration of such heterostructures is still at an early stage and usually confined to monolayers. We have grown heterostructures of Bi_2_Se_3_/C_60_ with exceptional crystallinity. Electron energy loss spectroscopy (EELS) reveals significant hybridisation of *π* states at the interface, despite the expectation for only weak van der Waals interactions, including quenching of 2D plasmons. Momentum-resolved EELS measurements are used to probe the plasmon dispersion, with Density Functional Theory predictions providing an interpretation of results based on interfacial charge dipoles. This work provides growth methodology and characterization of highly crystalline TI/molecular interfaces that can be engineered for plasmonic applications in energy, communications and sensing.

**Figure:**
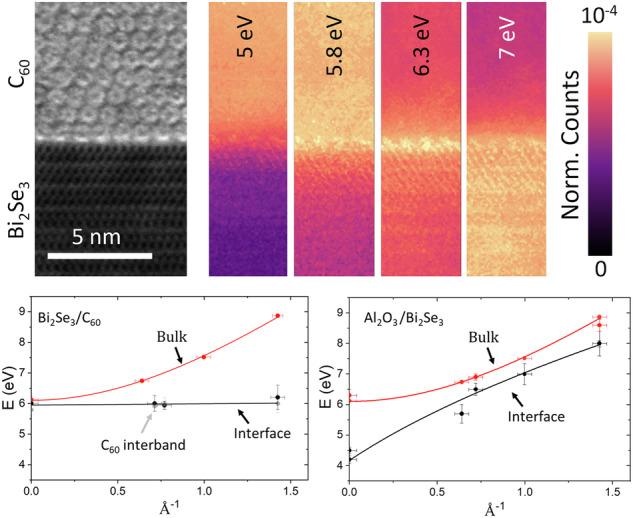

## Abstract (web summary)

Topological insulators offer promising potential for nanoscale, high-frequency devices, yet controlling surface plasmon properties remains challenging. Here, the authors grow Bi_2_Se_3_/C_60_ heterostructures with exceptional crystallinity, using electron energy loss spectroscopy and density functional theory to reveal significant *π* state hybridization and quenching of 2D plasmons.

## Introduction

A major barrier to the development of practical plasmonic devices is electronic scattering, which limits conduction efficiency, particularly in conventional metallic films^1^. However, low dimensional systems including graphene offer high mobility and low scattering rates, making them attractive for the development of sensing and communications applications^2–4^. Topological insulators are especially interesting as low dimensional plasmonic materials for operation at THz, optical and UV frequencies^5–8^ because of their topologically protected surface states (TSS). These arise from band inversion in the bulk^9^ and can support Dirac Plasmon modes^10^. Such collective excitations of two-dimensional (2D) Dirac states can exhibit suppressed scattering, efficient spin transport and, theoretically, spin-charge separation effects at plasmon frequencies due to spin momentum locking, a feature that may enable spintronic-photonic integration^11,12^.

Practical TI-based plasmonic devices will require a means of tunable control of 2D plasmons. One approach is to dope the surface with impurities to create a 2D electron gas (2DEG)^13^. For example, depositing Rb on freshly cleaved Bi_2_Se_3_ is observed to create a 2DEG with significant Rashba splitting^14^. However, these dopants are extremely reactive, so the effect is unstable outside ultra high vacuum. A more robust alternative is the use of thin films of organic molecules and dyes,^15–17^ which form hybrid surface states due to interactions between molecular *π* electrons and *π* states on the surface of the TI. Molecular thin films can support reversible charge transfer to an underlying TI, providing a route to tune the surface properties^18^. In particular, heterostructures of TIs, fullerenes and metallofullerens have been studied using ARPES^19–21^ demonstrating flat bands and modified Rashba coupling, but these studies are limited by penetration depth and thus cannot map effects across an interface. STEM-EELS has been used extensively to study TI thin films and heterostructures with 2D materials^22–24^ but has not been applied to TI/molecular heterostructures. An understanding of the hybrid interactions at TI/molecule interfaces is therefore vital for the rational design of hybrid materials and devices.

Characterisation of TI heterostructures is complicated by the accessibility of the surface. Electrical or THz characterisation can struggle to distinguish interface and bulk effects except at very low temperatures^25^. STEM-EELS provides a useful probe of localised surface effects where interfacial electronic structure can be probed with sub-nm resolution. STEM-EELS can detect plasmon excitations above 1 eV which arise from interband excitations as well as surface plasmon polaritons in the THz regime. In particular, the 2D *π* plasmon mode observed in both topological interfaces^24^ and 2D materials such as graphene^26^ provides a sensitive probe of the *π* electron states of the Se plane that terminates a Bi_2_Se_3_ film^27^. Hybrid surface *π* plasmons in both graphene and Bi_2_Se_3_ heterostructures have been used to create extremely efficient UV photodetectors^28,29^. In Bi_2_Se_3_, the *π* plasmon mode has been observed in free-standing films and shown to correspond to the presence of a 2D surface state, but has not been measured in a TI/molecular heterostructure^24^. Extending this methodology to TI heterostructures allows us to investigate how the *π*-states are modified by the presence of a molecular overlayer and investigate materials for gate-tuneable topological plasmonics.

## Results

### Sample Preparation

**Fig. 1:**
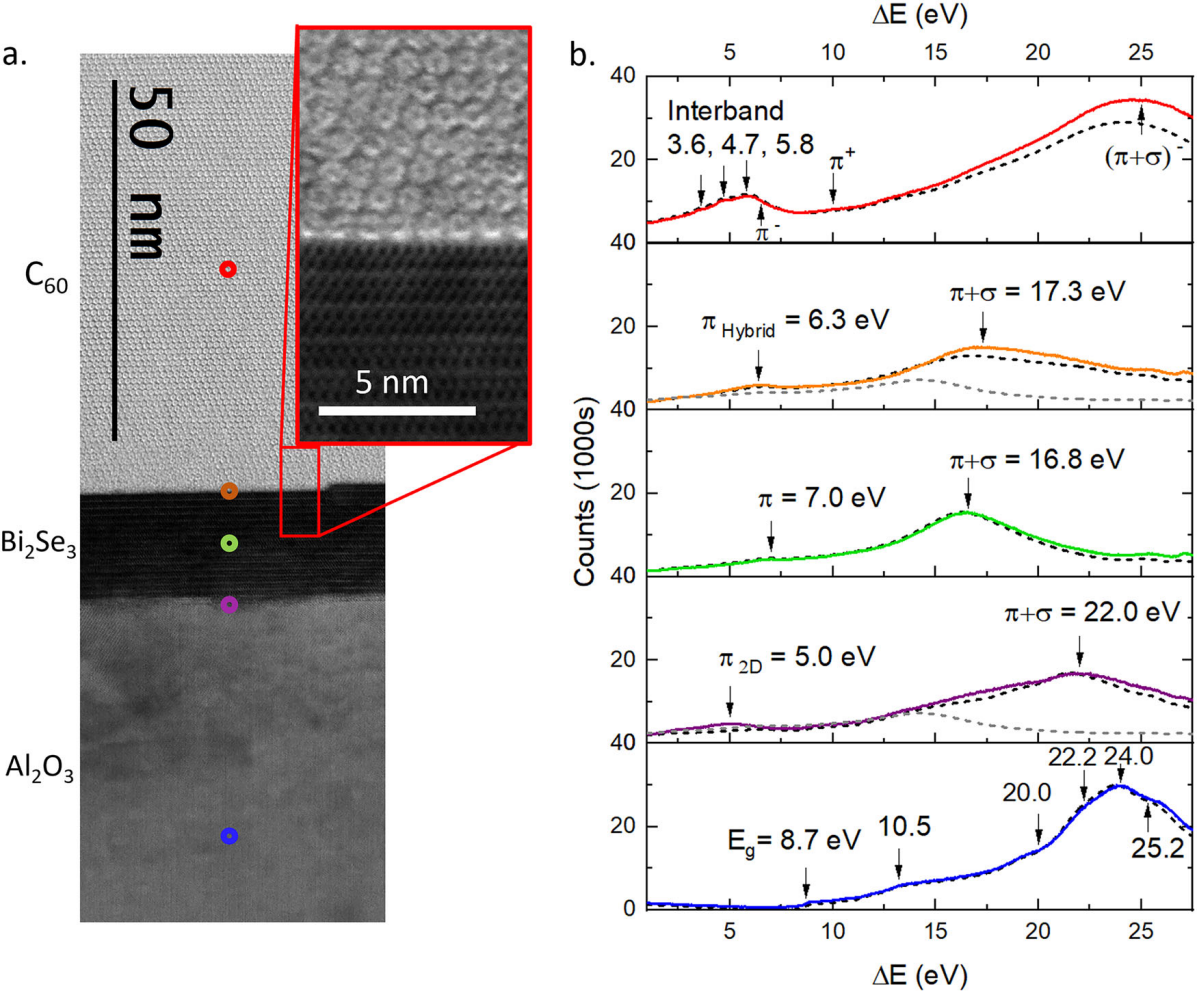
Cross-sectional STEM and layer spectra. **a** Cross-sectional STEM image of the sample lamella, showing approximate positions for the STEM probe for each spectrum. The inset shows high resolution image of the interface with individual fullerene columns clearly visible. **b** EELS spectra collected from C_60_ (red), C_60_/Bi_2_Se_3_ interface (orange), bulk Bi_2_Se_3_ (green), Bi_2_Se_3_/Al_2_O_3_ interface (purple) and bulk Al_2_O_3_ (blue), with key features marked. Analytical models of the EELS spectra, derived from empirical dielectric functions, are marked as black dotted lines. Models of the expected spectra for a vacuum interface are marked as grey dotted lines. These models are described in more detail in the Methods.

C_60_ films form an ideal, stable molecular overlayer for the study of Bi_2_Se_3_/molecule heterostructures due to their high electron affinity and chemical robustness. Highly crystalline, pure C_60_ films are robust to STEM characterization where most organics would be susceptible to beam damage. A region of the interface between Bi_2_Se_3_ and continuous films of highly crystalline C_60_ is shown in Fig. 1a. We have found that C_60_ forms highly ordered crystals on TI surfaces at very low deposition energy, producing atomically sharp interfaces that are free from structural defects and stable to degradation (see Methods). C_60_ was chosen for its high electron affinity, resulting in significant charge transfer from the TI interface which will form a surface charge dipole and modulate the UV plasmon response^19^.

Bi_2_Se_3_/C_60_ hybrid structures were grown in a multi-functional molecular beam epitaxy (MBE) system under continuous ultra high vacuum (UHV). 15 QL (quintuple layers), just over 15 nm thickness, of Bi_2_Se_3_ were deposited onto a c-plane sapphire substrate using a self regulating growth method. Detailed electronic characterisation of material produced from this system and the growth methodology are outlined in previous works^30^ and in Supplementary Fig. S1. C_60_ was then deposited using a low-temperature Knudsen cell, to a thickness of  ~ 80 nm. Cross-sections were extracted and thinned to  ~35 nm using a Focused Ion Beam (FIB), further details of which are provided in Methods. The films that we have grown show extraordinary crystallinity for a hybrid molecular film, to the extent that individual fullerene columns can easily be resolved. Figure 1a illustrates a cross-sectional STEM image of the TI and C_60_ layers, with further images presented in methods and Supplementary Fig. S2. EELS measurements suggest the C_60_ to be of high purity without detectable oxygen content. The films also have a remarkably low defect density for a van der Waals bonded molecular film. The diffraction pattern of the C_60_ is dominated by sharp spots, indicative of high crystallinity. Geometric phase analysis highlights a low density of stacking faults, running diagonally through the film with spacing of order 20 nm. These typically nucleate at step edges in the underlying Bi_2_Se_3_/C_60_. This analysis is shown in Methods.

### EELS spectrum imaging

To study the sample’s plasmonic structure, we mapped the EELS spectra below 28 eV across a cross-section of the sample. Figure 1b shows spectra collected from the positions indicated in color in the STEM image of Fig. 1a. In the C_60_ film, Fig. 1b [red], a cluster of three peaks in the 4–6 eV energy range is known to correspond to interband transitions between the highest occupied and lowest unoccupied molecular orbitals (HOMO and LUMO, respectively): specifically, from the HOMO to LUMO+1 at 3.6 eV, HOMO to LUMO+2 at 4.7 eV and HOMO-1 to LUMO at 5.8 eV^31^. As observed previously^31,32^, the features at 6.8 eV, 10 eV and 25 eV correspond to the *π*^−^, *π*^+^ and (*π*+*σ*)^−^ plasmonic modes of C_60_, respectively. These are excitations of the induced charge on the shell of the molecule. The *π* and *σ* labels denote the orbitals excited^32^ while the  − / + labels indicate the anti-symmetric/symmetric modes, where charge oscillations on the inner and outer surfaces of the C_60_ cage are either out of phase or in phase^31,33,34^. The *π*^−^ plasmon at 6.8 eV overlaps with the LUMO interband transitions^35^. Other features, at 13, 14.6 and 17 eV, are generally attributed to different ionization states of the fullerene^33^.

In the bulk Bi_2_Se_3_, two excitations are observed, at 7 eV and 16.8 eV. In previous work, these are ascribed to *π* and *π* + *σ* plasmon modes respectively, relating to plasmonic excitations of *π* and *σ* bonds in the Se layers^24^. For the sapphire substrate, the band gap at 8.7 eV is clearly evident in Fig. 1b [blue], followed by interband transitions at 10.5 eV and 13.2 eV. These are in agreement with literature, while transitions at 20.0 eV, 22.2 eV, 24 eV and 25 eV have all been observed in Al_2_O_3_ crystals with small numbers of defects that here we attribute to the focused ion beam sample preparation^36,37^.

It is clear that the two interfaces have different spectral characteristics to those of bulk materials or vacuum interfaces. Typically, an interfacial plasmon will depend on the dielectric functions of the surrounding materials, and the sample’s response can be considered to arise from a coupling of excitations in both materials due to the transmitted electron beam and its image charge. In order to distinguish the bulk and interfacial contributions, each spectrum is compared with an analytical model, shown by dotted lines in Fig. 1b. The model employs a retarded-field approach described previously (see also Methods)^38,39^ and uses empirically-determined dielectric functions. Since these spectra were calculated using bulk dielectric functions, we can isolate those excitations that are distinctive of surface and interfacial electronic structures not present in the bulk. In the spectral region of interest here, around the energy of the *π* plasmon below 10 eV, significant differences between model and experiment are observed at both interfaces. At the interface between the C_60_ and Bi_2_Se_3_, there is a single peak at 6.3 eV. This excitation lies between the C_60_ interband transition peak at 5.8 eV and the Bi_2_Se_3_ bulk *π* plasmon at 7 eV and does not match the energy of either the C_60_ or Bi_2_Se_3_ bulk plasmons. Thus, we label it a hybrid *π* - plasmon to reflect that it emerges only at the hybrid interface. At the interface between the Bi_2_Se_3_ and Al_2_O_3_, a distinct 2D *π* plasmon mode is evident, first appearing within 2QL of the interface. This excitation has also been observed at Bi_2_Se_3_/vacuum interfaces^24^, and can be excited in an aloof position well into the substrate, appearing in the Al_2_O_3_ band-gap more than 4 nm from the interface^40^.

**Fig. 2:**
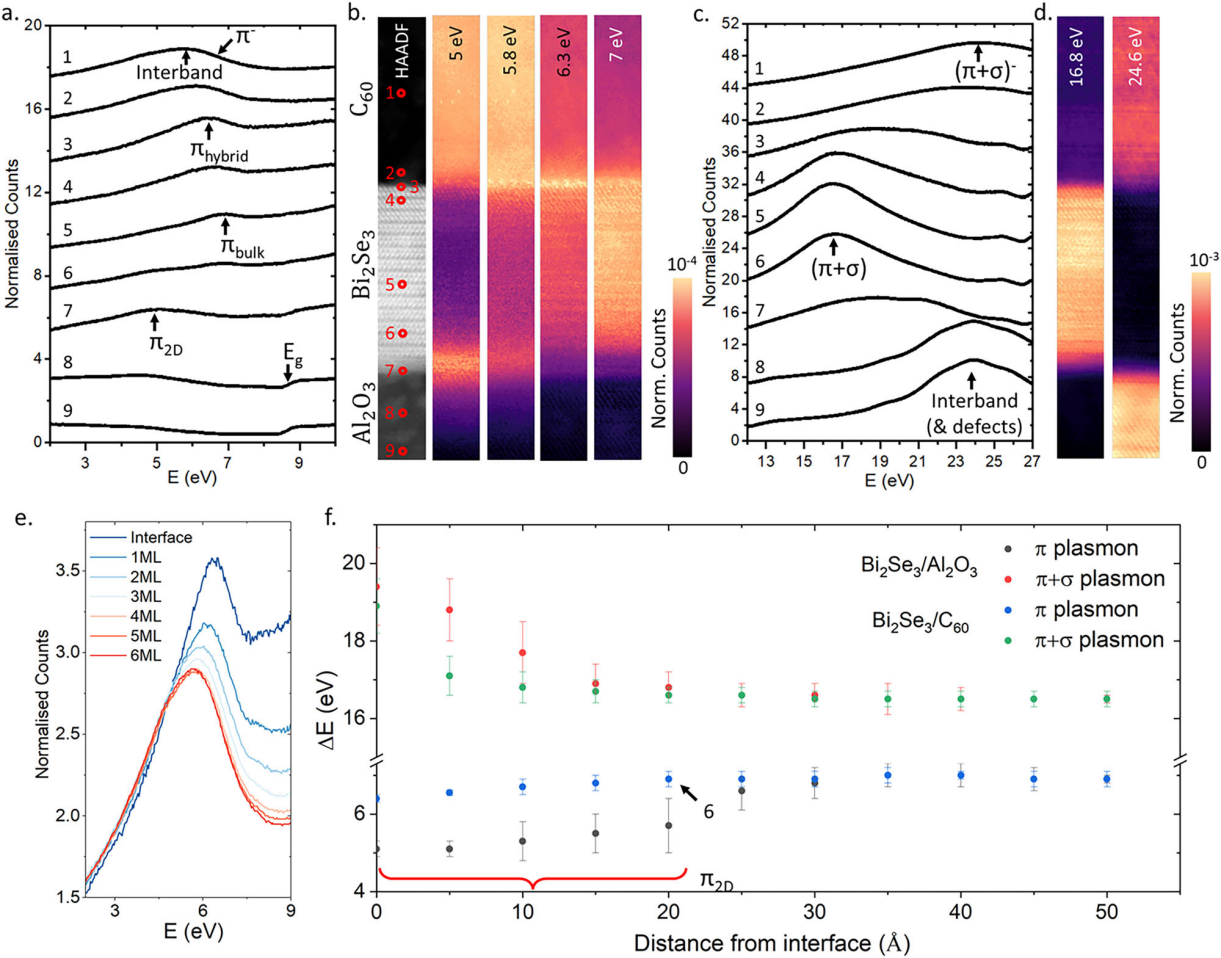
Low-loss EELS spectra and maps. EELS spectrum images collected across the two interfaces of Al_2_O_3_/Bi_2_Se_3_/C_60_. **a** EELS spectra collected from the region of the *π* plasmon with the STEM probe at the positions marked 1-9 in (**b**). The important features in each spectrum are indicated. **b** HAADF and spectral maps of a 3 × 30 nm region of the sample. Each spectral map shows the spectral density over the given energy window, normalised to the zero loss. **c** EELS spectra in the region of the *π* + *σ* plasmons located with the STEM probe at the positions marked 1-9 in (**b**). **d** Spectral density across the same region as (**b**). within the specified energy ranges. **e** Spectra in the region of the *π* - plasmon recorded at the top of each monolayer of the crystalline C_60_ overlayer showing the recovery of a bulk spectrum. **f** Energy of the *π* and *π* + *σ* plasmon modes in Bi_2_Se_3_ with distance from the interfaces with peak energy error. The data point at the position marked 6 in (**b**). is indicated.

Figure 2 shows in more detail the region of the *π* plasmon across a 3 × 30 nm region of the sample. The spectrum labels 1-9, Fig. 2a, correspond to the points marked on the HAADF image in Fig. 2b, which shows intensity maps across the same sample region over various energy ranges. All maps are normalised to the zero loss intensity and the *π* plasmon contribution is distinguished from the volume plasmons by fitting each spectrum with a pseudo-Voigt function and plotting the residuals. This removes artefacts in the spectral images caused by the tail of the volume plasmons increasing the apparent intensity of the *π* plasmon modes at the interfaces, detailed in Supplementary Fig. S3.

At 5 eV, the Bi_2_Se_3_ 2D *π* - plasmon mode is shown to be strongest at the Bi_2_Se_3_/Al_2_O_3_ interface and has non-zero amplitude in the band-gap of the sapphire. This mode decays exponentially into the substrate, indicating aloof excitation of the surface. The 2D mode also appears well into the Bi_2_Se_3_, first emerging in spectrum 6, 2QL from the interface. This raises interesting questions about the physical confinement of surface states in TIs. The C_60_ appears bright at this energy due to the tail of the broad interband transition peak in C_60_ highlighted in Fig. 1b, which extends well below 5 eV. There is, however, no evidence of a 2D plasmon at the Bi_2_Se_3_/C_60_ interface, indicating this behaviour is quenched by the C_60_. At 5.8 eV, the peak of the interband transition in C_60_ can be isolated, which is confined to the C_60_ film, overlapping somewhat with the 2D *π* plasmon.

6.3 eV corresponds to the expected energy of what we have labeled the hybrid *π* - plasmon mode. This range overlaps with both the bulk *π* - plasmon mode in Bi_2_Se_3_ and the interband excitations in C_60_, meaning there is a residual non-zero intensity in both bulk regions. The spectral intensity peaks at the Bi_2_Se_3_/C_60_ interface, which can also clearly be seen in spectrum 3 in Fig. 2a, showing that this is a surface effect. Interestingly, the contact points between individual fullerenes and the Bi_2_Se_3_ surface can be easily discerned as bright spots in the spectral map. This implies the 6.3 eV hybrid plasmon is not a 2D excitation, as might be expected, but an oscillation of the local charge dipole predicted in DFT. The 7 eV map highlights the bulk *π* - plasmon mode in Bi_2_Se_3_, which does not show this surface enhancement at either interface.

The *π* + *σ* volume plasmons in Bi_2_Se_3_ and C_60_ are shown in Fig. 2c, d, and show no surface amplification. The high surface intensity of the hybrid *π* - plasmon can be explained by Surface Enhanced EELS (SEELS). EELS is often more sensitive to molecular excitations at conducting interfaces due to resonant interactions between interband transition states and plasmon excitations in the surface^41^. As shown in Fig. 2e, the bulk spectrum of C_60_ is recovered two monolayers (1.64 nm) from the interface and, other than small changes in intensity, does not vary deeper into the C_60_. Further fitting and details of the C_60_ spectra are provided in Supplementary Figs. S2 and S5. This indicates strong surface confinement, and insignificant effects of delocalization on the spectra at the Bi_2_Se_3_/C_60_ interface.

The shift of the *π* and *π* + *σ* plasmon modes in Bi_2_Se_3_ are shown in Fig. 2f. At the Bi_2_Se_3_/C_60_ interface, the total redshift of the *π* - plasmon mode is just 0.7 eV, while the 2D *π* - plasmon mode seen at the Bi_2_Se_3_/Al_2_O_3_ interface is 2 eV below the bulk, identical to vacuum interfaces^24^. This suggests that it is not simply the difference in dielectric constant that alters the the Bi_2_Se_3_/C_60_ interface, and the C_60_ film has distinct effects on the TI surface states, which is supported by previous ARPES studies^21^.

**Fig. 3:**
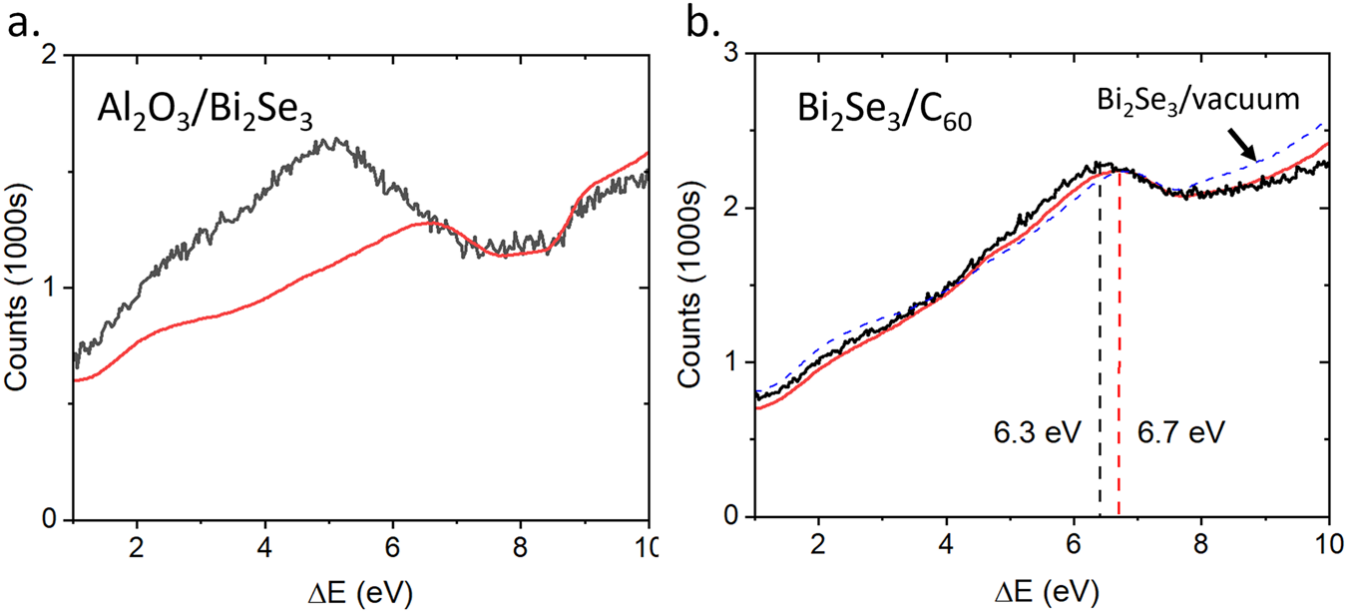
Comparison of *π* - plasmon data to model. Comparisons of the EELS model (red) and *π*-plasmon modes (black) at (**a**) the Al_2_O_3_/Bi_2_Se_3_ interface and (**b**) the Bi_2_Se_3_/C_60_ interface. The peak in panel a is not well reproduced by the model while panel b shows a slight peak-shift with respect to the model. Both aspects indicate that the observed surface features are not accounted for by red-shifted bulk plasmons. The blue dotted line in (**b**) shows an analytical model for a Bi_2_Se_3_ interface with vacuum.

A comparison of the *π*-plasmon region to the analytical model shows that the 2D *π* plasmon is not predicted by bulk dielectric functions as expected, Fig. 3a. The model of the Bi_2_Se_3_/C_60_ interface shows much closer agreement with experiment, as shown in Fig. 3b. The difference in the predicted redshift of the *π* plasmon at the Bi_2_Se_3_/C_60_ interface is likely because the model does not account for hybridisation of the C_60_ molecular orbitals with the TI surface *π* electrons. As has been previously reported in graphene^42^, *π* surface plasmons are very sensitive to charge doping and hybridisation. Therefore, the observed changes in the *π*-plasmon demonstrate that C_60_ can modify the surface properties, despite the weak absorption. If the degree of charge transfer could be modulated with an external bias or donor-acceptor complex, this modification might be externally tuned to control surface properties in TIs to engineer them for plasmonic applications such as UV detectors.

### Hybrid Plasmons

**Fig. 4:**
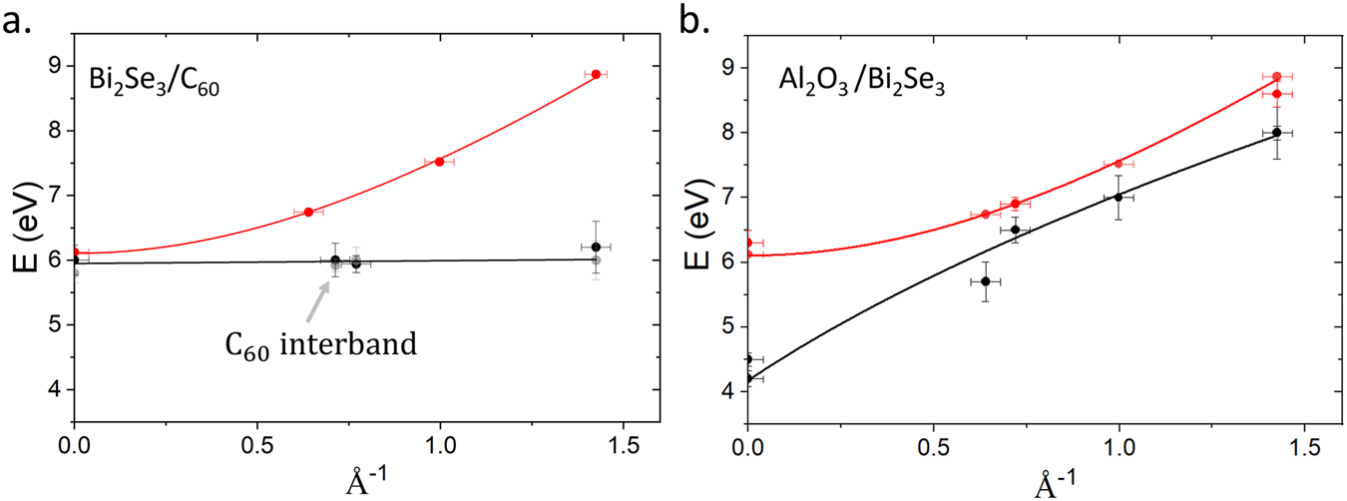
*π* - plasmon dispersion. **a** Dispersion for the bulk (red) and interface (black) *π*-plasmon mode obtained from QEELS spectra at the TI/C_60_ interface including momentum and peak energy error bars. The interface spectrum shows no dispersion and is within error of the C_60_ interband peak (grey points). **b** Dispersion for the bulk (red) and interface (black) *π*-plasmon mode obtained from QEELS spectra at the Al_2_O_3_/TI interface. Here, the surface obeys the $\sqrt{q}$ dispersion characteristic of a 2D plasmon excited via an interband transition^46^.

A deeper understanding of the effects of the C_60_ layer at the TI interfaces can be obtained by analysing changes in dispersion. Momentum-resolved EELS (QEELS) spectra from Bi_2_Se_3_ are shown in Fig. 4. Spectra were recorded with a momentum resolution of  ± 0.04 Å^−1^ over the range 0–1.43 Å^−1^ and in the *Γ* − *M* direction. In QEELS, all peaks appear shifted down in energy with respect to the unresolved EELS spectrum, with a difference in the energy of the q = 0 peak of  ≈ 300 meV. This is because integration over the larger, 22 mrad aperture used for STEM-EELS is weighted towards higher Q in comparison to the 2.4 mrad aperture used for QEELS (the correction is detailed in the Supplementary Fig. S4).

Plasmon peaks were fitted with pseudo-Voigt functions at each momentum with peak energy and peak width as free parameters (details of the fitting approach can be found in the Supplementary Fig. S5). In bulk C_60_, the three interband transitions were not observed to disperse with momentum, as expected for resonant modes. The *π*-plasmon also showed minimal dispersion, which is expected due to its resonant behavior and the presence of the C_60_ plasmon band-gap at 6.8 eV^43^. Bulk Bi_2_Se_3_ followed the well known bulk plasmon dispersion relation:

1 $${\omega }^{2}={\omega }_{p}^{2}+\frac{{q}^{2}{c}^{2}}{\varepsilon },$$

Where ${\omega }_{p}^{2}$ is the bulk plasma frequency ${\omega }_{p}=\sqrt{n{e}^{2}/{\varepsilon }_{0}m}$. However, the hybrid *π* - plasmon shows almost zero dispersion at the Bi_2_Se_3_/C_60_ interface, Fig. 4a. At the interface between Bi_2_Se_3_ and Al_2_O_3_, the *π*-plasmon peak followed a 2D plasmon dispersion with a *q*^1/2^ dependence, as shown in Fig. 4b^44,45^.

Liou et al. proposed the dispersion of 2D *π*-plasmons in Bi_2_Se_3_ followed:

2 $${\omega }_{p}{(q)}^{2}=\beta +\gamma q,$$

where *β* is the single particle oscillator strength and *γ* = 2*πn*_2*D*_*e*^2^/*mε*. This is the same behavior observed in free standing graphene^44–46^. Upon fitting the dispersion data for the plasmon confined to the Bi_2_Se_3_/Al_2_O_3_ interface with equation (2), *β* was 4.14 ± 0.02 eV. The 2D electron density was obtained from *γ*. The effective mass of *π* electrons excited into the parabolic band in the *Γ* − *M* direction was 1.05 *m*_0_ obtained from DFT. The permittivity was estimated from Kramers-Krönig analysis of bulk data as *ϵ*_1_ = 2.35, giving the 2D electron density of the *π*-plasmon as 1.9 ± 0.1 × 10^14^cm^−2^. This value is close to the estimated number of *π* bonded electrons in the surface Se layer of Bi_2_Se_3_ which was calculated to be  ≈ 7 × 10^14^cm^−2^ while the total number of *π* + *σ* electrons in the surface Se layer was found from DFT to be 2 × 10^15^cm^−2^, meaning roughly one in three *π* bonded electrons in the surface Se layer contribute to the *π* plasmon in this geometry. Details of this calculation can be found in the Supplementary Fig. S6.

**Fig. 5:**
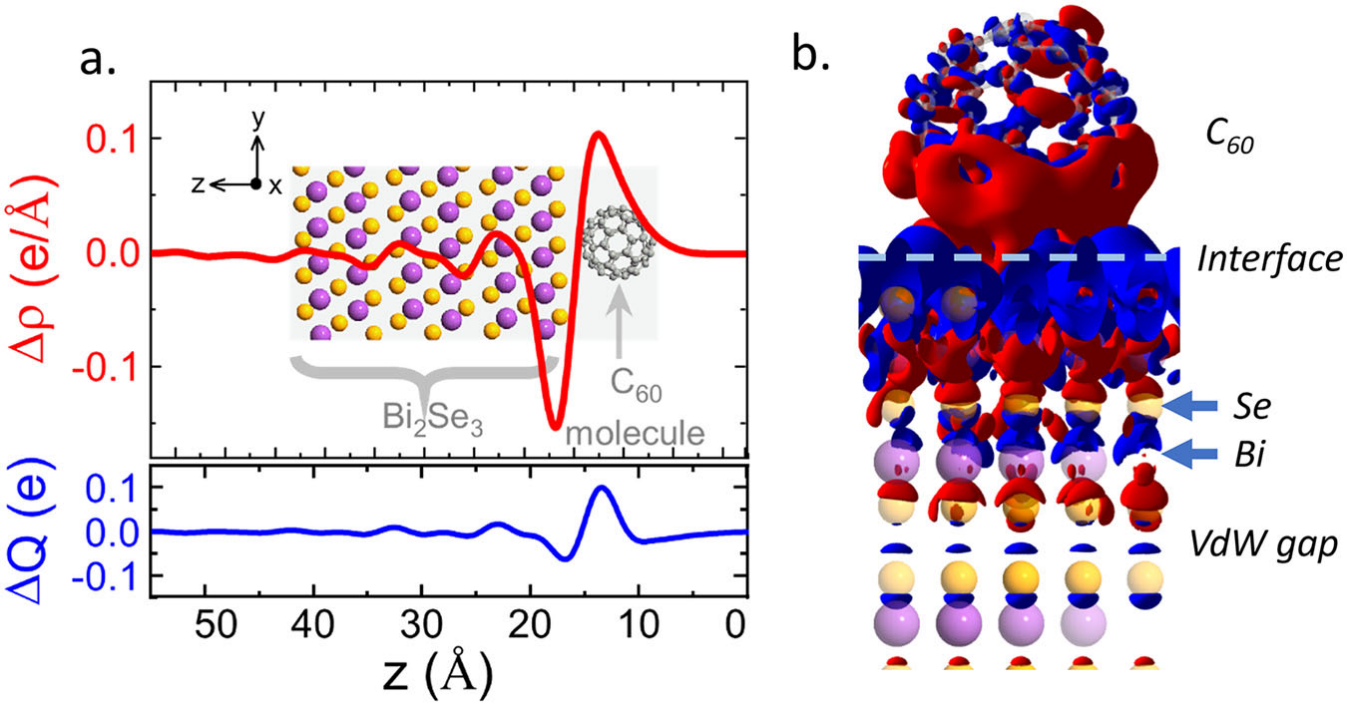
DFT models of interfacial charge transfer. **a** Charge density (red) and total charge (blue) changes across the TI/C_60_ interface, showing the formation of the interfacial dipole at the top-most QL. **b** Map of the TI/C_60_ interface with charge accumulation (blue) and depletion (red) shown.

Fitting the Bi_2_Se_3_/C_60_ interface with equation (2), *β* was measured to be 5.71 ± 0.01 eV. The *π* plasmon at this interface showed no dispersion within the available resolution, with the surface plasmon peak overlapping the expected C_60_ interband transition up to the edge of the first BZ. Using a relative permittivity of C_60_ at 6.3 eV of  ≈ 1^47^, the 2D electron density implied by equation (2) was 2.14 ± 0.01 × 10^13^cm^−2^, an order of magnitude lower than that of the TI/insulator interface. This is far lower than can be explained by the charge transfer predicted by DFT, Fig. 5a. The lack of dispersion and overlap with the C_60_ interband transition implies that the 2D *π* plasmon is suppressed at this interface, and the observed plasmon is a resonant excitation of the interfacial dipole shown in Fig. 5 b. The linewidth is clearly reduced between the bulk C_60_ spectrum and the Bi_2_Se_3_/C_60_ interface and the dominant interband transition peak at 5.8 eV is absent. A reduction in linewidth and suppression of interband transitions is consistent with theoretical models of electron doped C_60_^48^. This evidences that the hybrid *π* plasmon is fundamentally distinct from the 2D *π* plasmon reported at TI-vacuum interfaces. This 2D *π*-plasmon is strongly quenched at the molecular interface due to the hybridisation of the Se *π*-bonds with the C_60_*π* orbitals and resultant charge transfer.

While ARPES reports weak absorption of C_60_ onto Bi_2_Se_3_^21^, this study demonstrates that rich interactions can take place at such an interface, including the modification of surface potentials and the formation of hybrid states which may lead to emergent phenomena, including Rashba effects. This indicates the significant changes the C_60_ layer has induced in the surface through absorption, without significant structural changes to the underlying layers. These changes were only observed in highly crystalline C_60_ interfaces. Films grown at room temperature, with amorphised C_60_ showed bulk like dispersion at the interface, Supplementary Fig. S2. However, samples prepared in this manner showed no distinction between the different interband transitions in the bulk C_60_ film, with a single peak at 5.8 eV. This highlights the importance of high crystallinity in the properties of C_60_, which was also noted in ARPES by Latzke et al. ^19^

DFT simulations allow us to analyze the ways in which C_60_ modifies the Bi_2_Se_3_ surface. We find that the electron affinity of the C_60_ molecule leads to the formation of an interfacial dipole, with a total of 0.1 e transferred to each molecule, Fig. 5a, b. The formation of this dipole explains the suppression of the 2D *π* - plasmon and the emergence of the hybrid plasmon at 6.3 eV. The behaviour of the Bi_2_Se_3_/C_60_ interface is not well explained by the formation of a 2DEG due to band-bending, which is expected in many TI heterostructures and interfaces^13^, though DFT predicts band bending of  ≈100 meV at this interface.

ARPES studies of Bi_2_Se_3_/C_60_ interfaces indicated only weak interactions, requiring 2D molecules such as H_2_Pc to see strong effects on band-structure^21^. However, other studies of continuous molecular films showed features such as Rashba splitting even for much weaker surface coupling^15^. Since the surface *π* plasmon in 2D systems is extremely sensitive to changes in surface band structure^42^, the strong effect of C_60_ doping on the *π* plasmon dispersion in Fig. 4a is notable, since it implies that molecular layers can have significant effects on the surface state through charge transfer and hybridisation of the *π* electron states, which may be electrically tunable^49^. To explore the degree to which this surface can be electrically tuned, more work is needed, particularly utilising doped fullerenes such as Li@C60.

## Discussion

The engineering of interfaces in TI heterostructures is vital to the development of real world applications for topological insulators. Fullerenes present an interesting test system to explore hybrid plasmons at such engineered interfaces. We have demonstrated the deposition of highly crystalline films of Bi_2_Se_3_/C_60_ in which individual fullerenes can be imaged at the interface. We have shown that this interface has very different behaviour to the 2D *π*-plasmon measured at the TI/insulator or TI/vacuum interfaces, evidenced by a non-dispersive hybrid excitation confined to the interface, and quenching of the 2D *π* surface plasmon. The strong UV plasmon and potential for tunability provides a route to heterostructures for UV photodetectors which would be easier to grow and optimise than current TI based photodetectors, such as BSTS meta-materials^50^. The suppression of 2D carriers in the Bi_2_Se_3_/C_60_ interface is similar to that measured in optimized BSTS, but C_60_ coatings are much easier to optimize and control. There is also significant potential to use other organic dyes to optimize the optical properties of the surface. For example, heterojunctions of pentacene/Bi_2_Se_3_ show large photocurrents under blue light, believed to be due to interfacial potentials, which may involve similar effects to those measured in this work^51^. Using DFT, we have simulated the Bi_2_Se_3_/C_60_ interface as a model system to show how charge transfer gives rise to these emergent effects, which may be tuneable either via gating or doping. Together, this analysis provides important insight into the interactions between molecules and TI surfaces in the optical and UV spectrum, which can support the rational design of heterostructures for photodetectors and plasmonic devices. Future work should focus on exploring the effects of doping and gate control on this interface in order to actively modify the interfacial dipole and tune the properties of the surface.

## Methods

### Sample preparation

Bi_2_Se_3_/C_60_ hybrid structures were grown using a multi-functional molecular beam epitaxy (MBE) system, the Royce Deposition System, located at the University of Leeds. This system comprises linked chalchogenide MBE and organic MBE chambers, with a UHV transfer system that allows multi-step depositions to be performed under continuous UHV, eliminating the need for wet processing of organics, plasma cleaning or etching processes. TIs were deposited under a pressure of 10^−9^ mbar onto a c-plane sapphire substrate held at 230−240 ^∘^C. Growth was monitored using a RHEED system. The film grew progressively as a series of van der Waals coupled QL. Growth was solely determined by bismuth flux, with the Se flux at least 20 times higher to insure no chalcogenide vacancies form. This growth is self-regulating, as the sticking coefficient of the top surface changes as the QL builds up. The THz behaviour and structural qualities of these films are outlined in recently published work^52^. Once the film was grown and cooled, it was transferred under 10^−10^ mbar into an organics MBE. Here, the organic film was grown from a low temperature evaporation cell with the substrate held at  ~ 100 ^∘^C. The growth of the molecular films was monitored by a quartz balance. Pressure and trace gasses are monitored live by a ThorLabs RGA system, with total pressure not exceeding 10^−7^ mbar and O_2_ pressure not exceeding 10^−10^ mbar.

**Fig. 6:**
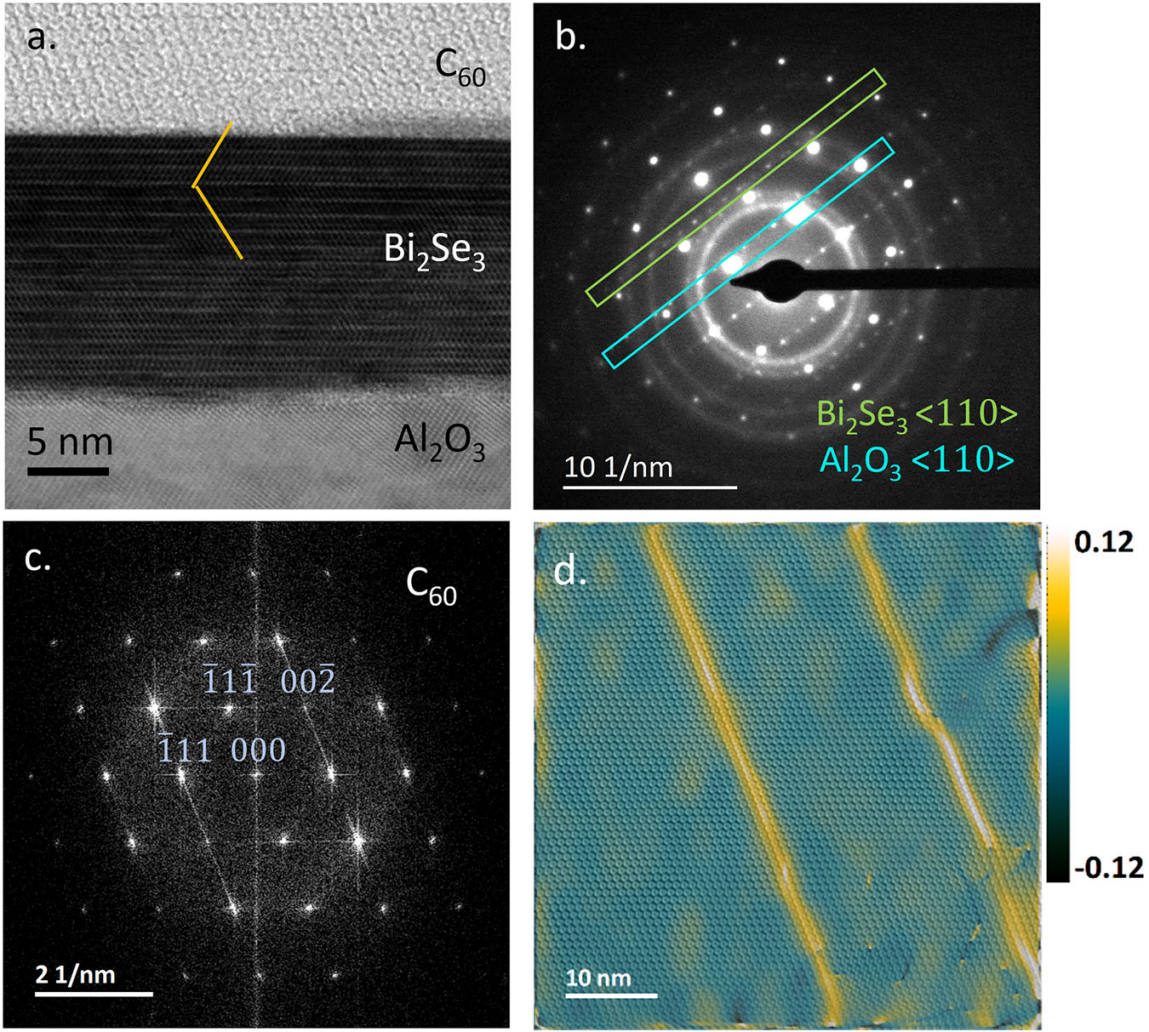
STEM analysis of sample structure. **a** BF STEM image of the heterostructure. There is a twin plane 3QL from the surface. This common defect is often caused by surface strain and can be suppressed through substrate doping^62^. **b** TEM diffraction pattern and indexing of the Bi_2_Se_3_ film and substrate. **c** Fourier transform of a STEM image with indexing of the C_60_ film. **d** GPA showing strain in the C_60_ film, highlighting stacking faults that run parallel from the surface with spacing of 20 nm.

Electron transparent lamella of the samples were prepared by focused ion beam (FIB) lift-out techniques using a dual beam electron-beam/FIB instrument, a Thermo Fisher Helios Xe Plasma FIB. A 30 kV xenon beam was used to mill into the bulk with currents 6.7 nA and 1.8 nA to extract a section which was subsequently thinned to a 35 nm thickness using a current of 74 pA and polished with a 5 kV 47 pA ion beam. Initial TEM characterisation was carried out at 200 kV to confirm the thickness and crystallinity of the samples before imaging in STEM. No direct epitaxial ordering was observed or expected between the film and substrate, as the surface of the sapphire is passivated and will form only weak van der Waals bonds with the thin film. The QLs grew with the c-axis parallel to the growth direction, such that the terminating surface of the last QL is exactly parallel to the substrate. The c-axis lattice constant is 28.6 Å while the quintuple layer thickness is 9.5 Å. Samples vary between 12 and 15 QLs thick across their surface, with single QL terraces forming at intervals of 20 - 50 nm. Details of the sample characterisation are shown in Fig. 6.

### EELS Model

To model the interfacial EELS spectra, each material’s response to the electric field of the STEM electron beam was first measured and used to derive a bulk dielectric function through a Kramers-Krönig analysis^53^. EELS spectra, *Γ*, between materials with dielectric functions *ε*_1_ and *ε*_2_ at a distance *b* from the interface in material 1 were then calculated using equation 3, where ${q}_{y}^{c}$ is a cutoff momentum defined by the spectrometer aperture, the electron velocity is *v*_*e*_, ${\alpha }_{i}^{2}=({(\omega /v)}^{2}+{q}_{y}^{2})-{\epsilon }_{i}{\omega }^{2}/{c}^{2}$ and fundamental constants have their usual symbols^38,39,54^. The cross-section thickness, *t*, was determined using a known mean free path, *λ* and measuring *t*/*λ* directly by EELS.^53^

3 $$\Gamma = 	 \frac{{e}^{2}}{2{\pi }^{2}{\varepsilon }_{0}\hslash {v}_{e}^{2}}\int_{0}^{{q}_{y}^{c}}\Im \left\{-\frac{1-{\varepsilon }_{1}{\left(\frac{{v}_{e}}{c}\right)}^{2}}{{\alpha }_{1}{\varepsilon }_{1}}\right.\\ 	 +\left.\frac{{e}^{-2{\alpha }_{1}b}}{{\varepsilon }_{1}{\alpha }_{1}({\alpha }_{1}+{\alpha }_{2})}\left[\frac{2{\alpha }_{1}^{2}({\varepsilon }_{2}-{\varepsilon }_{1})}{{\varepsilon }_{1}{\alpha }_{2}+{\varepsilon }_{2}{\alpha }_{1}}+({\alpha }_{2}-{\alpha }_{1})\left(1-{\varepsilon }_{1}{\left(\frac{{v}_{e}}{c}\right)}^{2}\right)\right]\right\}d{q}_{y}$$

### EELS methods

STEM-EELS measurements were carried out using the SuperSTEM3 instrument, a Nion UltraSTEM 100MC HERMES, incorporating a probe corrector and monochromator capable of producing a beam energy spread of 5 meV. Spectra were recorded with a beam energy of 100 keV and using Nion IRIS spectrometer, employing a 30 mrad convergence angle and a 22 mrad collection angle.

**Fig. 7:**
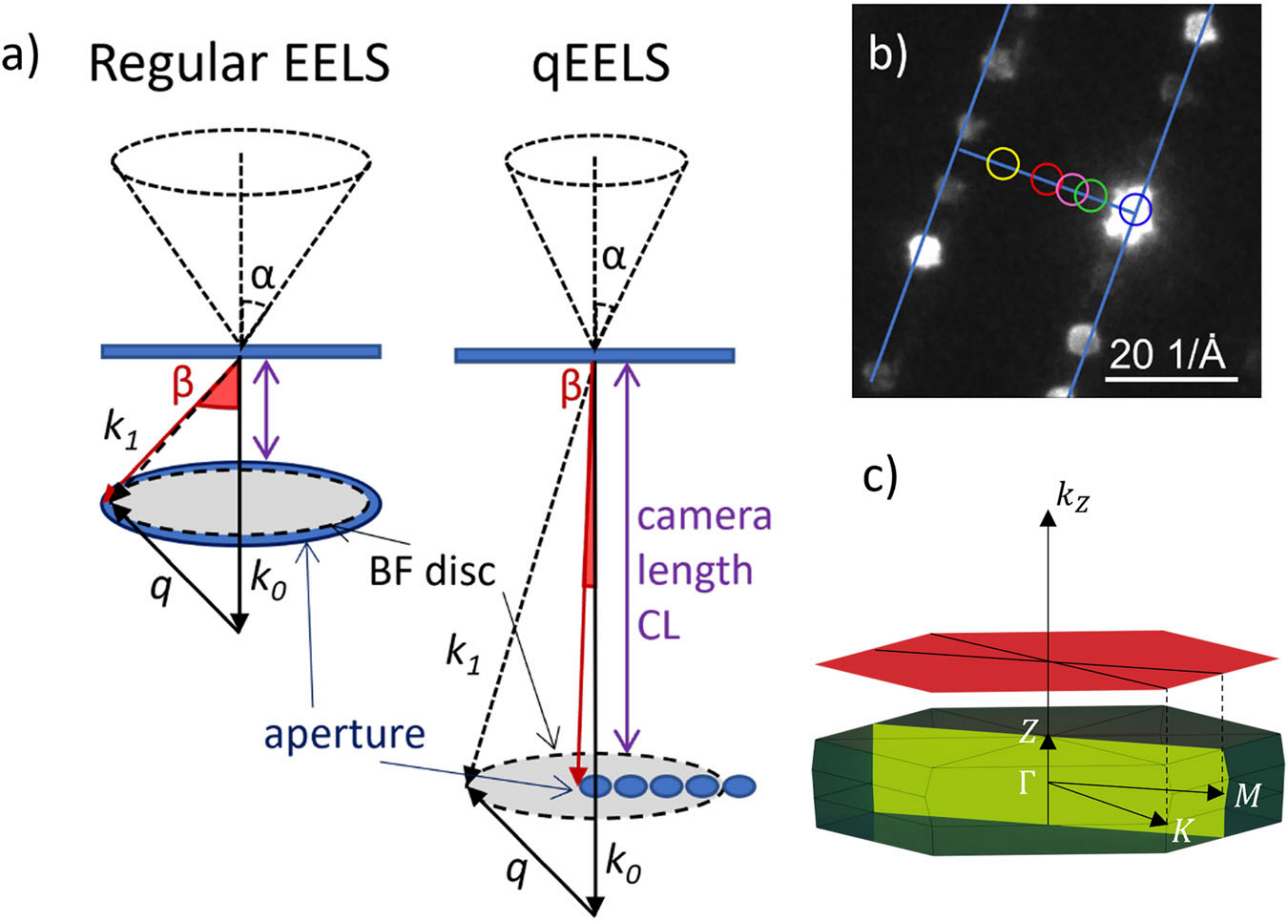
Momentum resolved EELS methodology. **a** Schematic with geometry of scattering in regular EELS and momentum-resolved EELS (qEELS) where *α* is the convergence angle, *β* is the collection angle, q is the momentum transfer (*q* = *k*_1_ − *k*_0_), q(*α*) is the radius of the bright field disc in reciprocal space and *Δ*q is the direction of the momentum acquired over. **b** Ronchigram of Bi_2_Se_3_ with the EELS aperture overlaid at the locations along the *ΓM* direction of the first Brillouin zone centred 0, 0.7, 1.0 and 1.4 A&ring; from the centre. **c** First Brillouin zone of Bi_2_ Se_3_ with high symmetry momentum directions indicated. The shaded yellow area represents a cross-sectional sample and the red shaded are a plane view sample.

The plasmon dispersion can be obtained from momentum-resolved EELS measurements which allow for the plasmon energy to be obtained at different momenta. This technique is an extension of standard STEM-EELS in which a focused electron beam is transmitted through a sample and collected by an EELS aperture into a spectrometer as shown in Fig. 7. In this case, instead of having a large EELS aperture to collect as many of the transmitted electrons as possible, a smaller aperture is used to further restrict the scattering angles, and hence momentum transfer range, accessed by the spectrometer^55^. This is achieved with a combination of increased camera length, smaller aperture and larger convergence angle. Using a larger convergence semi-angle increases the separation of the diffraction spots allowing for the aperture to only cover a small region of the Brillouin zone in reciprocal space. The scattering angle relates to the momentum transfer in the scattering process by

4 $${{\bf{q}}}(\theta )=4\pi \sin (\theta /2)/\lambda ,$$

where *λ* is the electron wavelength and *θ* is the scattering angle of transmitted electrons. The radius of the unscattered beam without an aperture, the bright field disc, in momentum space is calculated using *θ* = *α* the convergence angle. The momentum range over which the EELS spectrum is collected is found by *θ* = *β* the collection angle and the momentum resolution found using $\theta =\sqrt{{\alpha }^{2}+{\beta }^{2}}$, the root sums of squares of the convergence and collection angles. The resolution is determined by the relative size of the EELS aperture and diffraction pattern which results from both the collection and convergence semi-angles. The diffraction pattern is displaced with respect to the spectrometer aperture, along specific directions in momentum space to collect data at different momentum transfer values *Δq*. The high symmetry *ΓM* direction within the Brillouin Zone was determined using the diffraction pattern to navigate. EELS spectra were then decomposed to yield distinct contributions. A plot of the plasmon peak position versus the momentum transfer gave the plasmon dispersion relation.

### Density Functional Theory

To gain atomistic insight into the impacts of C_60_ molecules on the electronic structure of the Bi_2_Se_3_, first principles calculations were performed within the framework of density functional theory (DFT), as implemented in QuantumATK^56^. Linear combination of numerical atomic orbital (LCAO) basis set and generalised gradient approximation (GGA) with norm conserving pseudopotentials from PseudoDojo^57^ were employed. In order to obtain the Dirac cone surface states in the 2D energy-momentum relation, spin orbit coupling (SOC) through the use of fully relativistic pseudo potentials is included in the calculations. Brillouin zone integrations were performed over a grid of k points generated according to the Monkhorst Pack scheme^58^ with a density of approximately 10 k-points per angstrom. Energy cut off of 125 Ha has been considered for discretised grid and all structural relaxation was performed with the maximum force of less than 0.02 eV Å^−1^. Van der Waals correction to the GGA functional^59^ is considered to account for inter and intra molecular noncovalent of large range interaction. The slab in the supercell is infinite and periodic in the x and y directions (parallel to the slab surface) and is finite along the z direction (normal to the slab surface). The thickness of the vacuum region along the z-direction is larger than 20 Åto avoid any interaction between the periodic images of the neighbouring films. In the slab model calculation with asymmetric surface, an artificial macroscopic electrostatic field exists due to the periodic boundary conditions^60^. In order to avoid this artificial field in the Bi_2_Se_3_ with C_60_ molecule on the surface, we considered Neumann and Dirichlet boundary conditions at the C_60_ and Bi_2_Se_3_ sides of the slab, respectively, which provides an alternative approach for the dipole correction in the slab calculations^61^.

## Supplementary information

Supplementary Information

## Data Availability

Data is available from the corresponding author on request.
