# Supplementary material for: Investigation of hybrid plasmons in a highly crystalline Bi2Se3/C60 heterostructure using low-loss electron energy loss spectroscopy
Source: Commun Mater. 2025 Jul 29;6(1):166. doi: 10.1038/s43246-025-00886-0 (PMC12307225; doi:10.1038/s43246-025-00886-0)
Supplement: Supplementary file 1 — Supplementary Information [file 43246_2025_886_MOESM1_ESM.pdf]

**Supplementary Information for Investigation of hybrid plasmons in a highly crystalline $Bi_2Se_3/C_{60}$ heterostructure using low-loss electron energy loss spectroscopy**

Mairi McCauley, Lida Ansari, Farzan Gity, Matt Rogers, Joel Burton, Satoshi Sasaki, Quentin Ramasse, Craig Knox, Paul K Hurley, Donald MacLaren, Timothy Moorsom

### Section S1: $Bi_2Se_3$ quality

The quality of $Bi_2Se_3$ films was investigated using a combination of x-ray diffraction (XRD) and transport measurements, figure S4. The details of this material and its properties is further discussed in [2]. The films used in this work showed a half integer quantum hall coefficient indicative of the presence of a topological surface state at the $Bi_2Se_3$/vacuum interface which coexists with a 2DEG.

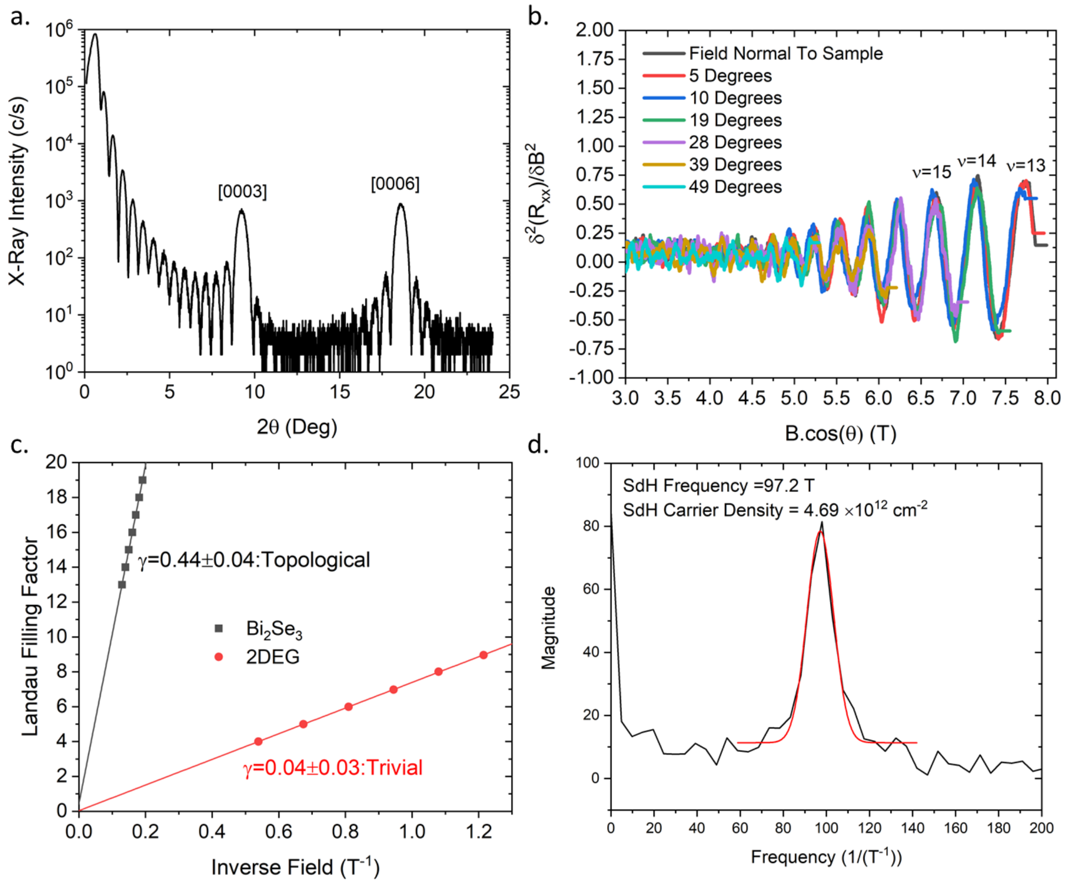


***Figure S1: $Bi_2Se_3$ characterisation.*** *a. X-Ray Diffraction of the fresh grown $Bi_2Se_3$ film. b. Quantum Hall measurements of a hall bar obtained from the same $Bi_2Se_3$ wafer showing Shubnikov de Haas oscillations with Landau levels labelled. c. Quantum Hall Coefficient fit of b showing half integer Quantum Hall Coefficient indicative of a topological surface state and its coexistence with a surface 2DEG. d. Fourier transform of the Shubnikov de Haas measurements showing the surface carrier density.*

### Section S2: C60 Quality

C60 layers were grown with high crystallinity by 100 C in UHV. XRD shows the characteristic [111] peak of crystalline C60 at 11 degrees, which is the main indicator of crystalline growth, figure S5a. However, growth at lower temperature still results in ordering in the z axis of the film but does not allow C60 columns to be resolved, figure S5b. Layers grown at this temperature do not appear to

have a fully reconstructed interface, with the C60 becoming amorphized in the last few ML. The plasmon dispersion for such layers is also quite different, showing something closer to bulk dispersion, but with a lower plasmon frequency. However, this dispersion relation should be viewed with some scepticism due to the amorphized nature of the C60. It cannot be clearly determined that the plasmon dispersion is at the interface, as the interface is not as well defined. Notably, a disordered surface does not change the energy of the hybrid mode, or the length scale over which it is screened, figure S5d. This indicates the chemistry of the surface has not changed and hybridisation of the C60 with the $Bi_2Se_3$ surface still occurs. The implication that a reconstructed surface is important for localisation of surface plasmons demonstrates that this effect is not solely a result of surface doping, but of modifications to the surface band structure.

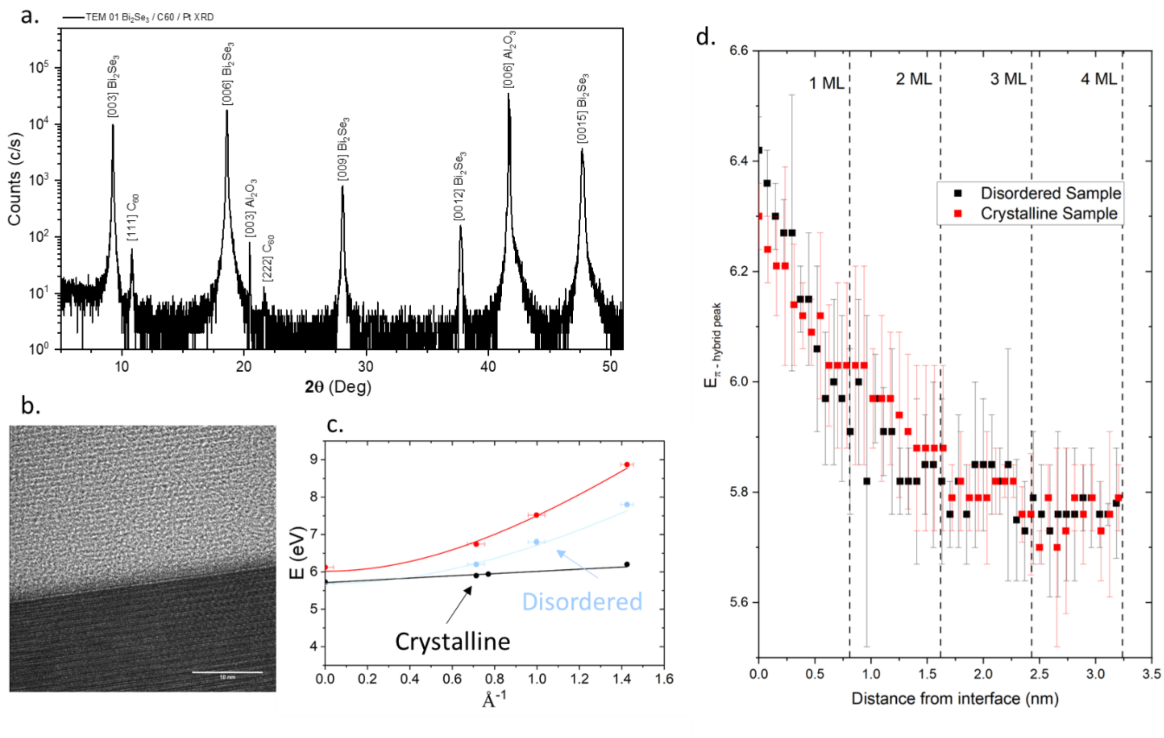


**Figure S2: C60 quality.** a. XRD of a TI/C60 bilayer showing the C60 structural peaks that indicate crystalline growth at 100 C. b. Cross-section of a layer deposited at room temperature. While the monolayer boundaries can still be seen, there are no resolvable C60 columns, meaning layers are not ordered. c. Comparison of the dispersion relation of the interface of the sample in b to data in figure 4 a. The disordered C60 (blue) shows a more bulk like dispersion relation. However, the disordered nature of the interface does not change the energy or screening of the hybrid plasmon d.

**Section S3: Spectral mapping**

In order to show the physical localisation of the different π plasmon modes, it is necessary to accurately deconvolve the spectrum. In particular the π+σ mode in the $Bi_2Se_3$ has a FWHM of almost 8 eV, meaning its tail can add a significant background intensity to the π plasmon. This can make the interface appear more intense than it actually is and create the impression of localised surface modes where they do not exist. In order to determine whether there actually is a hybrid surface mode, the volume plasmons (VP) were fit pixel by pixel in Digital Micrograph. The VP were then subtracted from the spectrum and the residuals plotted for the π plasmon range in Fig 3b.

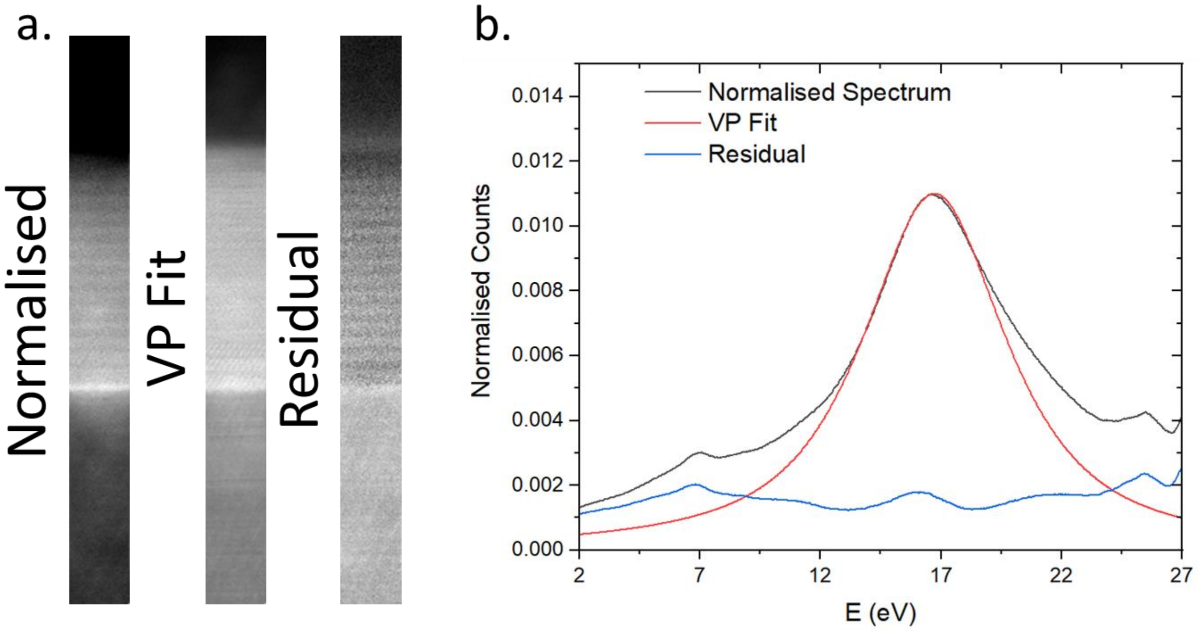


**Figure S3: Example of corrected spectra.** a. Three steps of correcting the spectra to correctly show the localisation of the 7eV bulk $Bi_2Se_3$ plasmon. The pixel-by-pixel volume plasmon fit is subtracted from the Normalised spectrum and the residual spectrum plotted. b. An example of the fit obtained from the $Bi_2Se_3$ layer.

**Section S4: Momentum Correction**

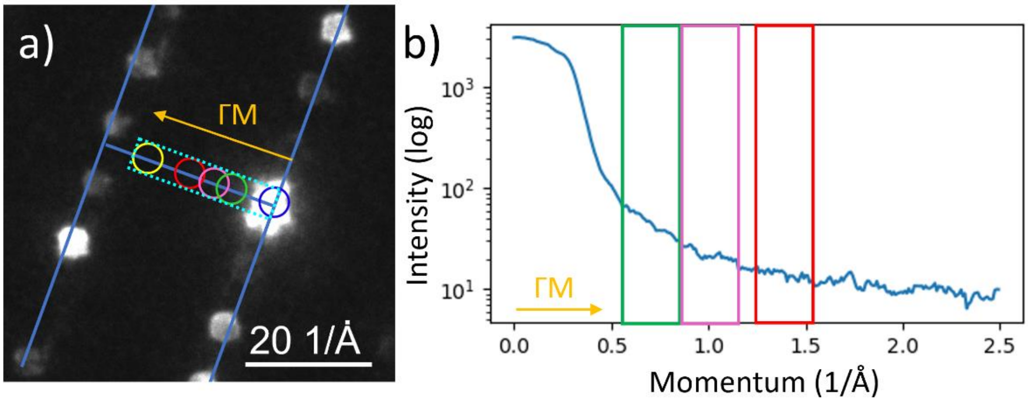


***Figure S4: Electron counts in off-axis qEELS spectra.*** *a) $Bi_2Se_3$ ronchigram with EELS apertures overlaid along the ΓM momentum direction. b) Integrated intensity, on a log scale, of the highlighted region of the ronchigram in a) along the ΓM direction starting from the centre of the central Bragg spot. At the first aperture position, 0.55 to 0.85, there was a larger contribution from electrons with lower momentum.*

Upon shifting to an off-axis position, the distribution of electrons collected by the aperture was skewed slightly to lower momentum due to the intensity of the central Bragg spot decaying towards higher momentum, figure S3. This resulted in a slight shift in the average momentum in qEELS spectra at low momenta close to the central Bragg spot. The momentum value for the first dataset was therefore adjusted to account for this. In each spot, the weighted average momentum was found.

$$\bar{q} = \frac{\int_{q_1}^{q_2} q \times I(q)\, dq}{\int_{q_1}^{q_2} I(q)\, dq}$$

Applying this correction to subsequent higher momentum spectra had no impact on the momentum value within the noise.

**Section S5: qEELS Peak Fitting**

Momentum-resolved EELS spectra were fitted at each momentum using a linear least-squares fit of several peaks as shown in figure S2 using HyperSpy [1]. The low energy region of each q-EELS spectrum was fitted between 1 and 12 eV to limit free parameters and prevent overfitting.

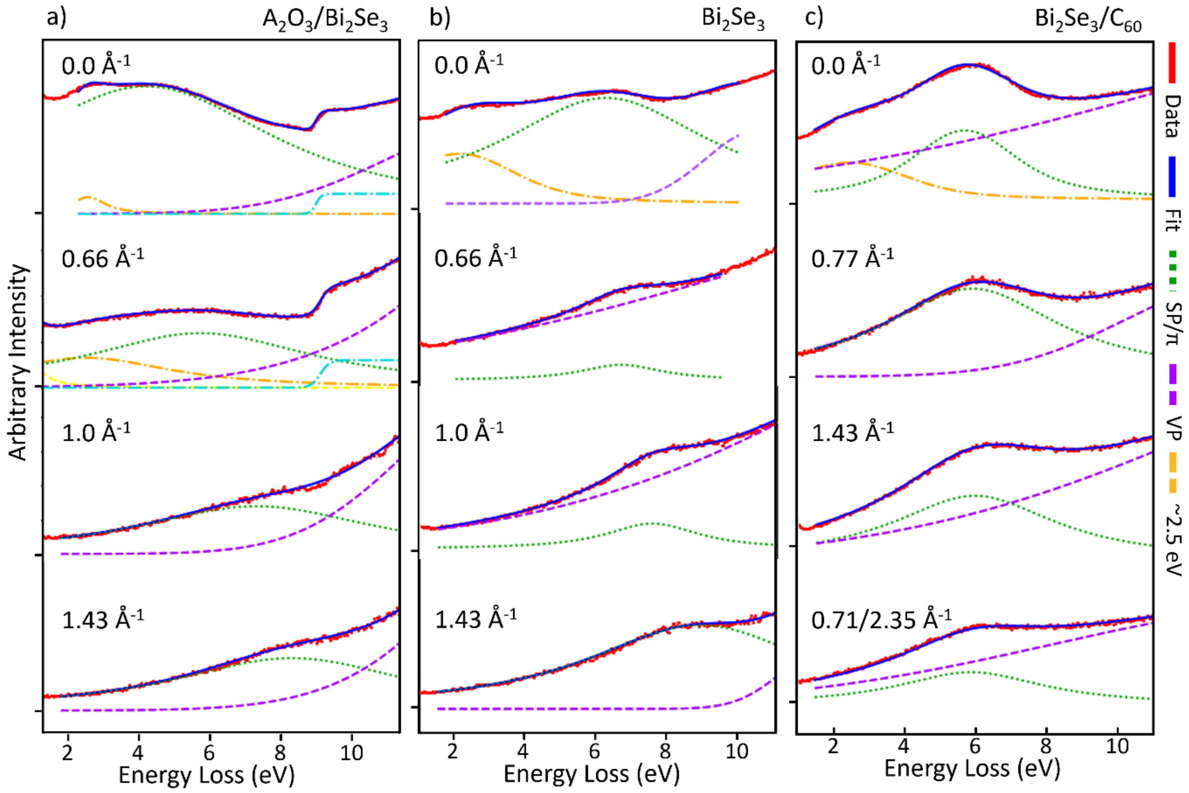


***Figure S5: Momentum-resolved EELS spectra with fitted peaks.*** *Fitted spectra at a) the $Al_2O_3/Bi_2Se_3$ interface, b) bulk $Bi_2Se_3$ and c) $Bi_2Se_3/C_{60}$ interface at each momentum. The red trace is the experimental data, the blue line is the combined fit, and the dotted lines are individual fit components. In each spectrum, the higher energy peaks were approximated using a gaussian peak shown in purple. The π plasmon was fitted using a pseudo Voigt peak indicated in green. At the $Al_2O_3/Bi_2Se_3$ interface, a logistic function was used to fit the $Al_2O_3$ bandgap, shown in cyan, with its parameters fitted in bulk $Al_2O_3$ and fixed in the interface fit. An additional peak at 2.5 eV was included in low momentum spectra indicated in orange.*

The bandgap in bulk $Al_2O_3$ was fitted with a logistic function, equation 1, at each momentum with parameters of slope, $c$, and centre position, $x_0$, fitted within reasonable boundaries and the area, $a$, allowed to be free. The slope of this function became less steep as the bandgap changed from direct to indirect with increasing momenta. This fitted bandgap was added as a component in the interface fit with its area as the only free parameter for low momentum spectra as in figure S2a.

$$f(x) = \frac{a}{1 + \mathrm{e}^{-\mathrm{c}(\mathrm{x}-\mathrm{x_0})}} \quad (1)$$

Alongside the bandgap, a large Gaussian function was included to approximate the volume plasmons and higher energy features. Surface and bulk plasmons were fitted using pseudo-Voigt functions, a convolution of a Gaussian and Lorentzian function with a free area parameter and bounded centre, gamma (Lorentzian) and sigma (Gaussian) values. Spectra at a momentum value of 0.0 Å were fitted with an additional peak at 2.5 eV which decreased in intensity, becoming insignificant, at subsequent momenta. The energy of this peak was fixed between 2-3 eV with a smaller intensity than other peaks.

**Section S6: Carrier Density Calculation**

The 2D carrier density was obtained from the equation:

$$\omega_{2D}^2 = \alpha + \gamma q$$

However, because details of this equation are often left ambiguous in literature, it is useful to detail the calculation we have used. The square of the 2D plasmon frequency can be plotted against the angular wave-vector, q, to directly obtain the gradient:

$$\gamma = \frac{2\pi n_{2D} e^2}{m\varepsilon}$$

in which $n_{2D}$ is the 2D electron density, $e$ electron charge, $m$ the effective mass and $\varepsilon$ the relative permittivity. In literature, $n_{2D}$ is sometimes referred to as the "carrier density" but this quantity is distinct from the number of free carriers in either the TSS or 2DEG. For interband excitations, this number refers to the density of excited valence electrons in the relevant band, in this case π bonded electrons in the $Bi_2Se_3$ surface. [3,4] The effective mass of π electrons excited into parabolic bands was determined from DFT to be 1.05 $m_0$ though it should be noted this calculation is unperturbed, and thus is an approximation. The relative permittivity can be estimated via Kramers – Krönig analysis, though this is again an approximation from bulk behaviour. Both $Bi_2Se_3$ and $Al_2O_3$ are estimated to have relative permittivity between 2-5 at 6 eV. [5, 6]

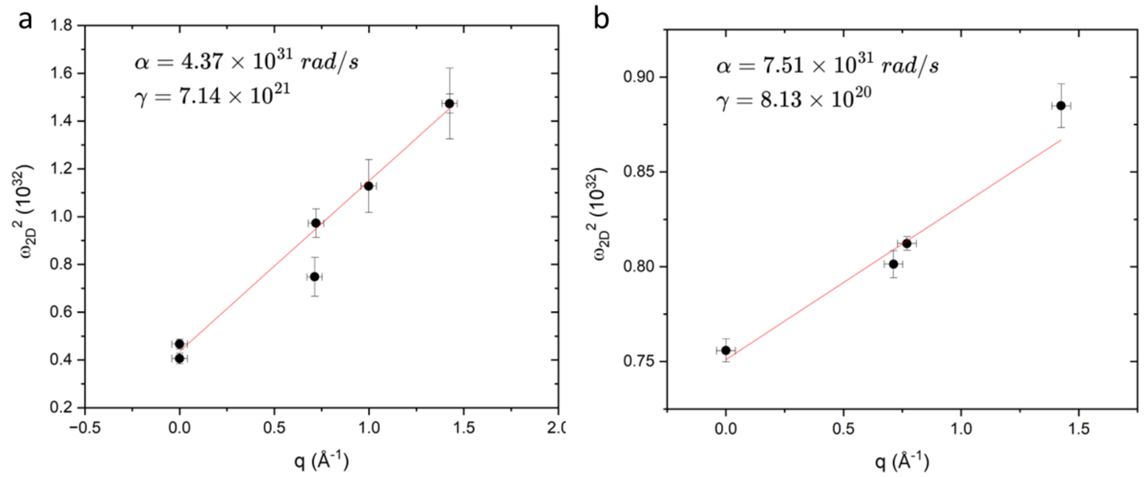


**Figure S6: Plots of plasmon dispersion.** a) Plasmon dispersion plotted on corrected axes showing the obtained values of α and γ at the $Bi_2Se_3/Al_2O_3$ interface. b) The same plot and fit for the $Bi_2Se_3/C_{60}$ interface.

G J Shu et al extracted the number of excited electrons per atom, $n_{eff}$, for the bulk π plasmon. [7] Unlike graphene, it is not possible to show a clear plateau in $n_{eff}$ at the π and π + σ transitions that clearly identify how many electrons are involved in each excitation. However, at 7eV, they observed a small change in gradient at a value $n_{eff}$ = 1 and E = 7 eV, and a second change in gradient at $n_{eff}$ = 5 and E = 17 eV. They concluded that the π electrons contributing to the π plasmon arise from an unpaired electron in the $p_z$ orbital of the Se atoms at the VdW interface, while the π+σ electrons come from unpaired electrons in the $sp^2$ hybrid orbital. At the interface, this would mean each surface Se atom should contribute maximum one electron to $n_{2D}$ in the surface plasmon. With a lattice constant a = 4.156 Å, the electron density for surface π electrons contributing to the π surface plasmon is thus predicted at $6.7 \times 10^{14}\ cm^{-2}$.

[1] Francisco de la Peña, Eric Prestat, Vidar Tonaas Fauske, Pierre Burdet, Jonas Lähnemann, Petras Jokubauskas, Tom Furnival, et al. 'Hyperspy/hyperspy: V2.0'. Zenodo, 20 December 2023. https://doi.org/10.5281/zenodo.10412190.

[2] V Pistore, L Viti, C Schiattarella, E Riccardi, C S Knox, A Yagmur, J J Burton, S Sasaki, A G Davies, E H Linfield, J R Freeman, M S Vitiello. Adv. Optical Mater. 12, 2301673, 2024.

[3] M Nakayama, T Kato and K Ohtomi. Solid State Comm. 50, 5, 1984.

[4] A Nagashima, K Nuka, H Itoh, T Ichinokawa, C Oshima, S Otani, Y Ishizawa. Solid State Comm. 83, 8, 1992.

[5] A K Harman, S Ninomiya and S Adachi. J Appl. Phys. 76, 12, 1994.

[6] M Fang, Z Wang, H Gu, M Tong, B Song, Z Xie, T Zhou, X Chen, H Jiang, T Jiang and S Liu. Appl. Surface Science, 509, 144822, 2020.

[7] G J Shu, S C Liou, S K Karna, R Sankar, M Hayashi and F C Chou. Phys. Rev. B. 2, 044201, 2018.
